# Preparation Method Determines Valinomycin Organization and Phase Behavior in Charged Langmuir Monolayers

**DOI:** 10.1021/acs.jpcb.6c03977

**Published:** 2026-10-26

**Authors:** Amrita Chakraborty, Priyojit Das Gupta, Anabela Meier, Antara Roy, Saranya Pullanchery

**Affiliations:** Department of Chemistry, 634082 Texas A&M University, College Station, Texas 77843-3255, United States

## Abstract

Amphiphilic molecules are typically incorporated into lipid Langmuir monolayers by two distinct methods: co-spreading, where lipid and amphiphile are deposited simultaneously, and injection, where the amphiphile is introduced into the aqueous subphase beneath a preformed monolayer. Whether these methods produce equivalent molecular arrangements remains an open question. Using valinomycin as a model ionophore, we investigate this question across lipid monolayers bearing anionic, zwitterionic, and cationic headgroups. Surface pressure–area isotherms, fluorescence imaging, and vibrational sum-frequency generation spectroscopy together reveal that the two preparation methods produce fundamentally different molecular organizations. Cospreading yields a uniform, well-incorporated monolayer, while injection produces a kinetically trapped, spatially heterogeneous state in which valinomycin resides predominantly at the headgroup–water interface. The magnitude of these differences scaled with lipid headgroup charge, implicating specific interactions between lipid headgroups and the carbonyl-lined cavity of valinomycin as key determinants of the incorporation pathway. These findings establish that preparation history is a critical parameter governing molecular organization in mixed amphiphile-lipid monolayers.

## Graphical abstract

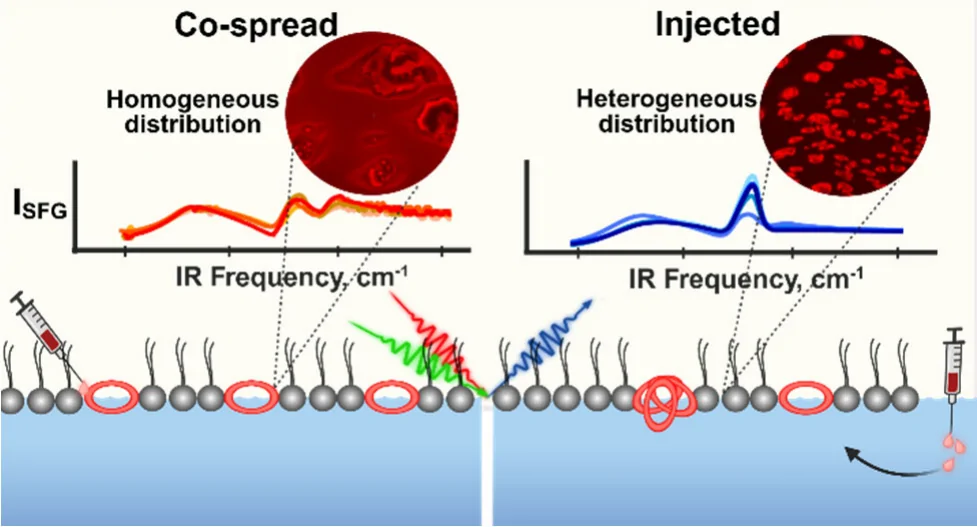

## Introduction

Interactions between amphiphilic molecules and lipid membranes are of fundamental importance in various biological and biomedical processes: Cationic antimicrobial peptides bind and disrupt microbial cell membranes, amphiphilic ionophores partition to membrane surfaces and mediate ion transport, and small molecule drugs must cross the membrane barrier to reach their intracellular targets.^1–9^ In each of these contexts, the ability of an amphiphilic molecule to bind and incorporate into a lipid membrane is the crucial first step in governing biological activity. Understanding the molecular details of amphiphile-lipid membrane association is therefore essential for both biological insight and rational design of membrane-active components.

Langmuir monolayers of lipids at the air/water interface have long served as model systems for investigating interactions between membrane lipids and their binding partners.^10–12^ Despite modeling only one leaflet of a biological membrane, Langmuir monolayers offer precise control over membrane composition and charge density, and the ability to independently vary lipid composition and phase behavior. These features enable detailed thermodynamic characterization that is difficult to achieve in bilayer systems.^13,14^ Two distinct preparation methods are routinely employed to incorporate amphiphilic molecules into such monolayers.^15–17^ In the cospreading method, lipid and amphiphile are dissolved together in a volatile organic solvent and deposited simultaneously onto the air/water interface, where they self-organize as the solvent evaporates. In the injection method, a lipid monolayer is first formed at the air/water interface, and the amphiphile is subsequently introduced into the aqueous subphase, from which it absorbs and penetrates the monolayer. These two methods yield complementary experimental observables and are chosen to match a particular biological or technological context. Cospread monolayers can be compressed to generate surface pressure- mean molecular area isotherms. Deviations of the measured mean molecular area from the ideal weighted average of the pure components reveal intermolecular interactions between the lipids and amphiphiles.^18,19^ Injection experiments enable surface pressure vs time measurements that report on the kinetics and barriers associated with amphiphile adsorption.^20,21^ Despite the widespread and context-driven use of both approaches, a critical question remains unresolved: do cospreading and injection produce equivalent molecular arrangements within the mixed monolayer?

This question has significant implications for interpreting and comparing results across different laboratories and research contexts. If both methods yield identical equilibrium structures, findings obtained by either approach should be directly comparable. If, however, the preparation pathway influences the final molecular organization -either as differences in mixing efficiency or kinetic barriers to amphiphile penetration- then studies employing different methods may report on fundamentally different states of the system. Resolving this question requires a model system for which both preparation methods are relevant, combined with a characterization approach capable of detecting molecular-level organizational differences.

Valinomycin is an ideal model system for addressing this question because its real-world applications naturally involve both preparation methods.^6,22,23^ This cyclic depsipeptide, produced as a secondary metabolite by *Streptomyces* bacteria, functions as a selective potassium ionophore.^24,25^ In technological applications, including ion-selective electrodes and liposomal formulations for controlled ion permeability, valinomycin is deliberately premixed with membrane components during fabrication,^26,27^ a process most accurately modeled by the cospreading method (Scheme 1A). In its native biological context, valinomycin must travel from the aqueous environment to a target membrane,^28,29^ a process best modeled by subphase injection (Scheme 1B). Early investigations of mixed valinomycin-lipid monolayer systems used the cospreading method with neutral and anionic lipids,^30–33^ but direct comparison between preparation methods using molecular-level characterization techniques has not been undertaken.

**Scheme 1:**
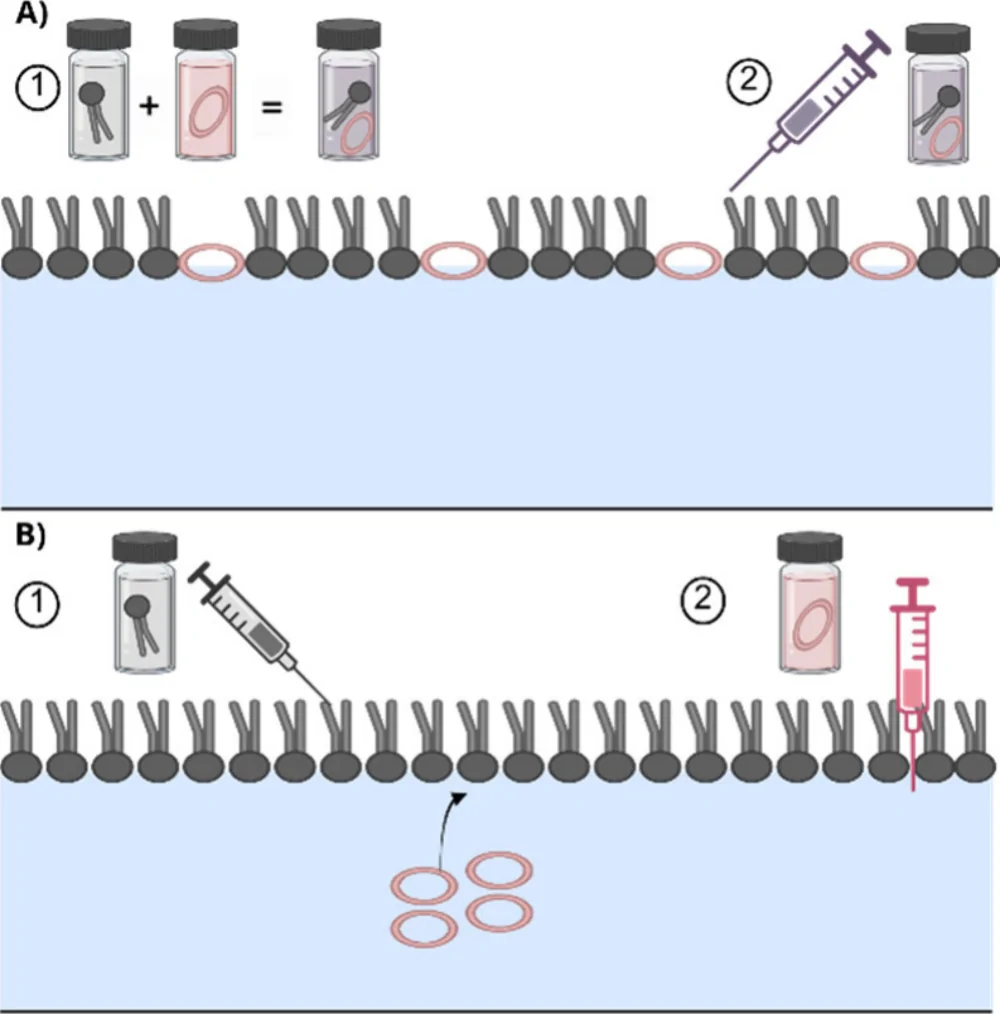
Illustration of the Two Methods of Incorporating Valinomycin into Lipid Monolayer^a^

a A) Cospreading: The two components are mixed together in a volatile organic solvent in (step 1) and co-deposited at the air/water interface (step 2). B) Injection: A lipid monolayer is first formed at the air/water interface (step 1), and valinomycin is subsequently injected into the aqueoue subphase (step 2).

Vibrational sum frequency generation (SFG) spectroscopy^34–38^ offers a uniquely powerful approach for this comparison. In SFG, a tunable mid-infrared beam and a fixed-frequency visible beam are overlapped on a sample surface. When the infrared frequency is in resonance with molecular vibrations at the interface, sum frequency photons are detected. Due to the symmetry selection rules governing this second-order nonlinear process, only anisotropically ordered molecular dipoles at the interface generate a sum frequency spectrum. SFG spectroscopy has proven to be a valuable tool in probing Langmuir lipid monolayer interfaces,^39–46^ and has been used to investigate the adsorption of amphiphilic solutes at lipid membranes.^47–59^ The surface selectivity of SFG spectroscopy makes it uniquely suited to determine whether different incorporation methods lead to different molecular organization, providing insights inaccessible to macroscopic thermodynamic measurements alone.

Herein, we investigate whether cospreading and injection produce equivalent molecular arrangements when valinomycin (Figure 1A) is incorporated into lipid monolayers bearing zwitterionic, positively, or negatively charged headgroups. Surface pressure area isotherms were used to characterize the thermodynamic behavior of the mixed monolayers and revealed method-dependent differences that also varied with lipid charge. Fluorescence imaging visualized the spatial distribution of monolayer lipid phases and their dependence on the preparation method. To understand molecular-level organization within these mixed monolayers, we employed vibrational SFG spectroscopy in the carbonyl, C–H, and O–H stretch regions, which provides spectroscopically distinct signatures for lipids and valinomycin. Together, these measurements demonstrate that the two preparation methods do not yield equivalent monolayers and suggest that preparation history encodes itself in the molecular organization of the mixed monolayer in a manner that depends systematically on the lipid headgroup charge.

**Figure 1:**
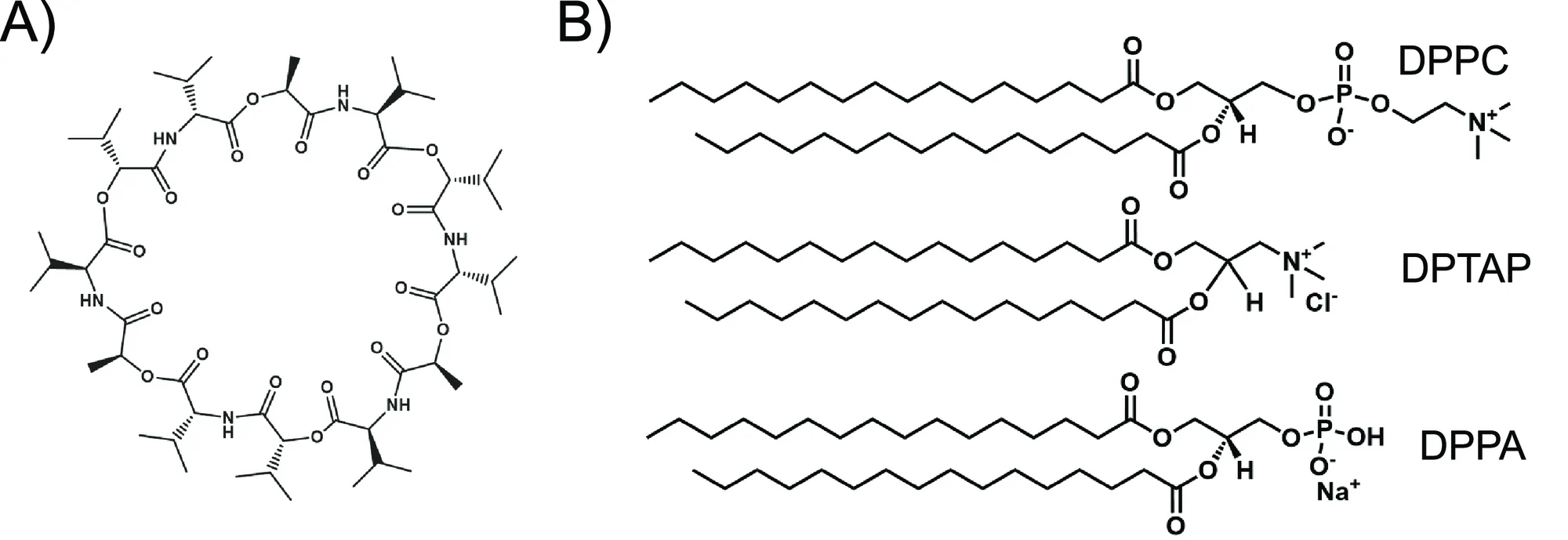
(A) Structure of valinomycin. (B) Structures of the three lipids used in the surface pressure - area measurements.

## Results

### Preparation Method and Charge-Dependent Phase Behavior

To determine whether cospreading and injection lead to different thermodynamic states, surface pressure–area isotherms of mixed monolayers formed at 4:1 lipid: valinomycin molar ratio (Experimental details in SI sections S1 and S2) were measured using lipids with three distinct headgroup charges: anionic 1,2-dipalmitoyl-*sn*-glycero-3-phosphate (DPPA), zwitterionic 1,2-dipalmitoyl-*sn*-glycero-3-phosphocholine (DPPC), and cationic 1,2-dipalmitoyl-3-trimethylammonium-propane (DPTAP) (Figure 1B).

Valinomycin itself is amphiphilic, partitions spontaneously to the air/water interface to form a Langmuir monolayer,^60,61^ and its surface pressure–area isotherm exhibits a single liquid expanded phase (Figure 2A, inset). An ideal two-dimensional mixture would have a mean molecular area equal to the weighted average of the pure components;^18^ calculated ideal-mixing curves (*A*_*mix*_ = 0.8 × *A*_*lipid*_ + 0.2 × *A*_*valinomycin*_) therefore serve as a baseline against which method-dependent deviations can be assessed. The deviations from ideal behavior for the two preparation methods were assessed using two complementary metrics: 1) the excess free energy of mixing (Δ*G*_*exc*_), a cumulative measure of nonideality integrated over the full compression (SI section S3), and 2) the local phase behavior near ∼ 30 mN/m, the monolayer–bilayer equivalent pressure, at which monolayer phase behavior is thought most directly relevant to lipid bilayers.^13,62^ The local phase behavior was captured by the presence or absence of a distinct valinomycin collapse transition and quantified by the compressional modulus.

**Figure 2:**
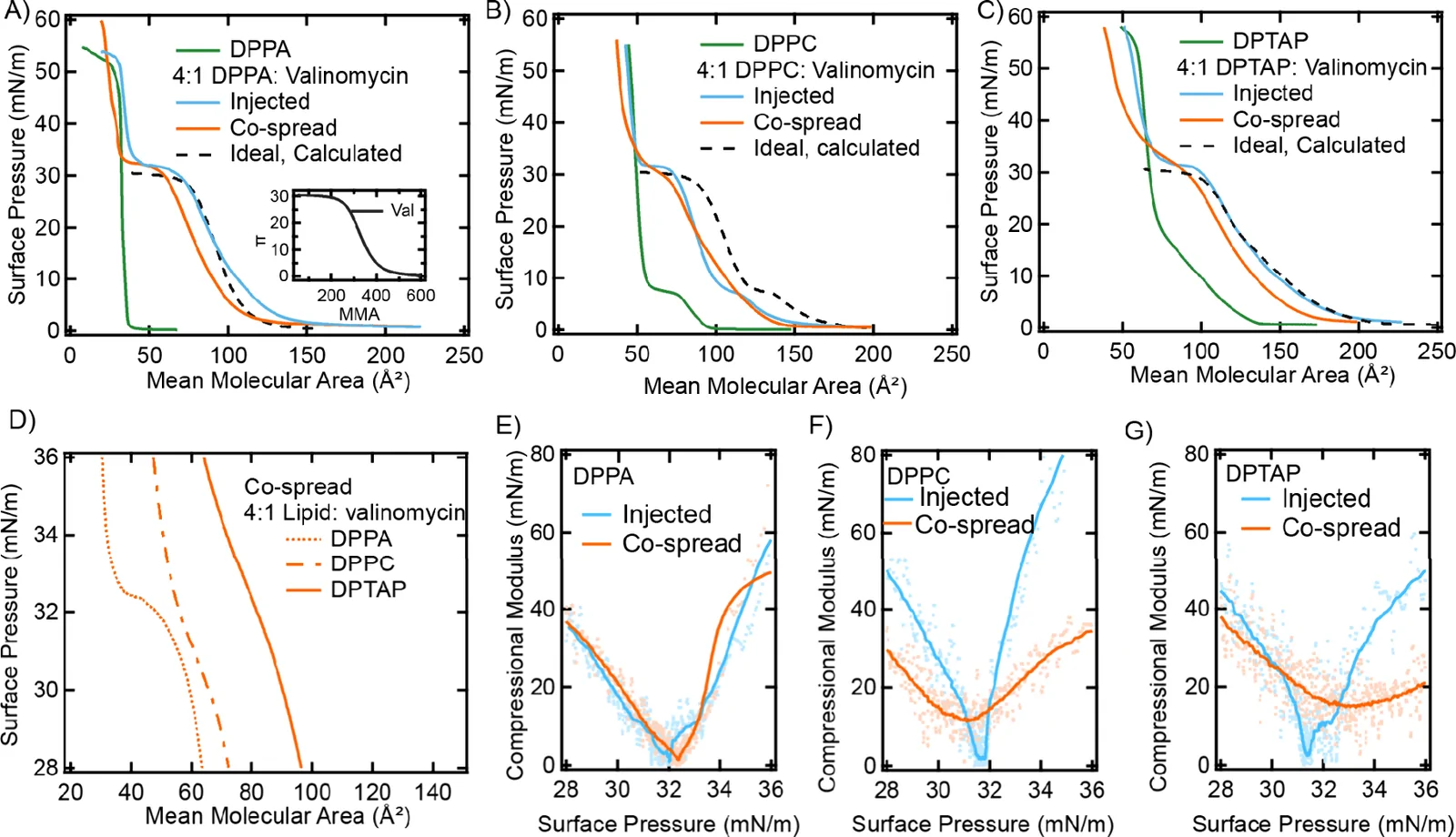
Effect of preparation method on surface pressure–area isotherms and compressibility of valinomycin-lipid monolayers. (A-C) Surface pressure-mean molecular area isotherms of pure lipids (green), 4:1 lipid: valinomycin mixtures prepared by injection (cyan) and cospreading (orange), and calculated ideal mixtures (black dashed) for DPPA (A), DPPC (B), and DPTAP (C). Inset in (A) shows the isotherm of pure valinomycin. (D) Expanded view of cospread isotherms from (A-C) in the surface pressure range of 28–36 mN/m, highlighting differences in phase transition behavior. Line styles distinguish lipid type: DPPA (dotted), DPPC (dashed-dotted), and DPTAP (solid). (E-G) Compressional modulus as a function of surface pressure for injected (cyan) and cospread (orange) monolayers of DPPA (E), DPPC (F), and DPTAP (G), calculated over the surface pressure range shown in (D). Scatter points represent raw data; solid lines are running averages as a guide to the eye.

For anionic DPPA monolayers, the calculated ideal mixing curve (Figure 2A, black dashed) exhibited a horizontal plateau around ∼ 32 mN/m that is characteristic of the valinomycin phase behavior.

If components were to mix ideally or be completely immiscible, the measured isotherm of the mixed monolayers would match this ideal curve.^63^ Note that the ideal mixing calculations in Figure 2 (A-C) were performed only at surface pressure values at which both pure component isotherms have defined mean molecular area values. The excess free energy of mixing (SI, Table S1) was smallest in magnitude among the three cospread systems for DPPA (−1.32 to –1.60 kJ/mol, versus –2.48 to –2.93 kJ/mol for DPPC and –2.08 to –2.21 kJ/mol for DPTAP) and, uniquely among the three lipids, changed sign under injection (+0.78 to +0.87 kJ/mol). This sign difference, together with the small magnitude of both values relative to the other two lipids, indicates that DPPA couples only weakly with valinomycin regardless of preparation method. This conclusion is also supported by the similarity in shape of the cospread and injected isotherms, with each retaining the characteristic horizontal plateau of valinomycin collapse (Figure 2A, cyan and orange). Together, these observations suggest that valinomycin retains its own monolayer character when mixed with DPPA, whether the peptide is present during spreading or introduced later from the subphase.

Zwitterionic DPPC monolayers (Figure 2B) exhibited a liquid expanded phase between 75 and 95 Å^2^/molecule, a coexistence region between ∼ 57–75 Å^2^/molecule, and a liquid condensed region between ∼ 44–55 Å^2^/molecule, consistent with previous reports.^64^ The ideal mixing calculation (Figure 2B, black dashed) predicts that without significant valinomycin-lipid interactions, the lipid molecules in the mixed monolayer would retain the liquid-expanded to condensed transition around ∼ 7.5 mN/m, and the valinomycin collapse around ∼ 32 mN/m as distinct features. The excess free energy calculations (SI, Table S1) revealed overall attractive interactions between DPPC and valinomycin with both preparation techniques, indicated by the similar negative Δ*G*_*exc*_ values. However, Δ*G*_*exc*_ is cumulative, so similar total values do not rule out local differences near the valinomycin collapse pressure: key differences between the two preparation methods emerge upon closer inspection of the isotherm shape around a surface pressure of 30 mN/m. Injection of valinomycin beneath a DPPC monolayer produced an isotherm (Figure 2B, cyan) that retained the horizontal collapse plateau characteristic of the ideal mixture. This indicates that valinomycin collapses as a separate phase rather than mixing with the lipid. Above 32 mN/m, the injected mixture isotherm converged with pure DPPC. In contrast, the cospread monolayer (Figure 2B, orange) exhibited a markedly different behavior: a single liquid expanded phase at surface pressures below ∼ 28 mN/m, followed by a less horizontal, sloped transition. This indicates that upon cospreading, valinomycin is incorporated into the monolayer even above its collapse pressure.

Pure DPTAP monolayers undergo a liquid expanded to condensed transition between ∼ 70–82 Å^2^/molecule (Figure 2C, green). DPTAP: valinomycin monolayers formed by injection (Figure 2C, cyan) closely resembled an ideal mixture. These monolayers also had a near-zero (−0.19 to –0.01 kJ/mol) Δ*G*_*exc*_, (SI, Table S1) consistent with DPTAP and valinomycin either mixing ideally or remaining immiscible under injection. The cospread monolayer exhibited negative Δ*G*_*exc*_ values (−2.08 to –2.21 kJ/mol), indicating attractive interactions between DPTAP and valinomycin. Additional evidence for these attractive interactions comes from the cospread isotherm behavior above 36 mN/m. The cospread DPTAP: valinomycin monolayers can be compressed to lower mean molecular areas than the pure DPTAP, suggesting the formation of lipid-valinomycin complexes that adopt more compact molecular conformations than either pure component. These results implicate the interactions between trimethylammonium groups on DPTAP and the carbonyl-lined cavity of valinomycin as a key driver of method-dependent behavior. A similar trend also appears for DPPC (Figure 2B), but to a lesser extent.

All cospread monolayers exhibited significant deviations from the horizontal collapse plateau behavior of valinomycin. To quantify the effects of the two incorporation methods near the monolayer–bilayer equivalent surface pressure,^13,62^ we focused on the isotherm phase behavior in the 28–36 mN/m range. Across this range, all three charged lipid: valinomycin monolayers formed by injection exhibited the horizontal plateau characteristic of valinomycin collapse. However, cospread monolayers showed a clear lipid-charge dependence (Figure 2D): the slope of the isotherm around ∼ 32 mN/m followed the order: DPPA < DPPC < DPTAP. These differences were quantified using the compressional modulus, $K=-A{\left(\frac{d\pi }{dA}\right)}_{T}$ , where *A* is the mean molecular area and π is the surface pressure. A compressional modulus approaching zero indicates a monolayer with minimal resistance to lateral compression, which is characteristic of noninteracting molecules that freely rearrange upon compression without raising the surface pressure. Figure 2E-G shows the compressional moduli for injected and cospread monolayers of each lipid. For DPPA: valinomycin (Figure 2E), both preparation methods yielded moduli approaching zero around 32 mN/m, consistent with noninteracting behavior of valinomycin with DPPA in this surface pressure range. DPPC (Figure 2F) and DPTAP (Figure 2G) exhibited progressively larger deviations, with DPTAP showing the maximum difference between preparation methods near the monolayer–bilayer equivalent surface pressure.

### Preparation-Dependent Phase Heterogeneity in DPTAP Monolayers

Because DPTAP monolayers exhibited the largest isotherm slope differences between preparation methods in the surface pressure range 28–36 mN/m, these monolayers were investigated by fluorescence imaging. All monolayers were labeled with Texas Red 1,2-dihexadecanoyl-*sn*-glycero-3-phosphoethanolamine (TR-DHPE), a fluorescent probe that partitions into the liquid expanded phase of the monolayer.^65,66^ Pure DPTAP monolayers at approximately 30 mN/m exhibited micron-scale domain coexistence (Figure 3A), with bright regions corresponding to liquid expanded domains (where the probe preferentially partitions) and the dark regions corresponding to liquid condensed domains. Most of the monolayer existed as liquid condensed regions, as expected for lipids with gel to liquid phase transition temperatures at or above room temperature.^62,66,67^ The phase behavior of DPTAP monolayer remained the same for a 30 min observation window (Figure 3B). This two-phase coexistence of DPTAP was not significantly altered by the injection of valinomycin into the subphase beneath a preformed DPTAP monolayer (Figure 3C), with the large areas of the monolayer still exhibiting similar domain behavior. In contrast, monolayers prepared by cospreading DPTAP: valinomycin mixtures (Figure 3D) exhibited a more spatially homogeneous fluorescence signal from Texas Red DHPE relative to Figures 3A-C, indicating that the cospread monolayer is more homogeneous compared to the injected monolayer.

**Figure 3:**
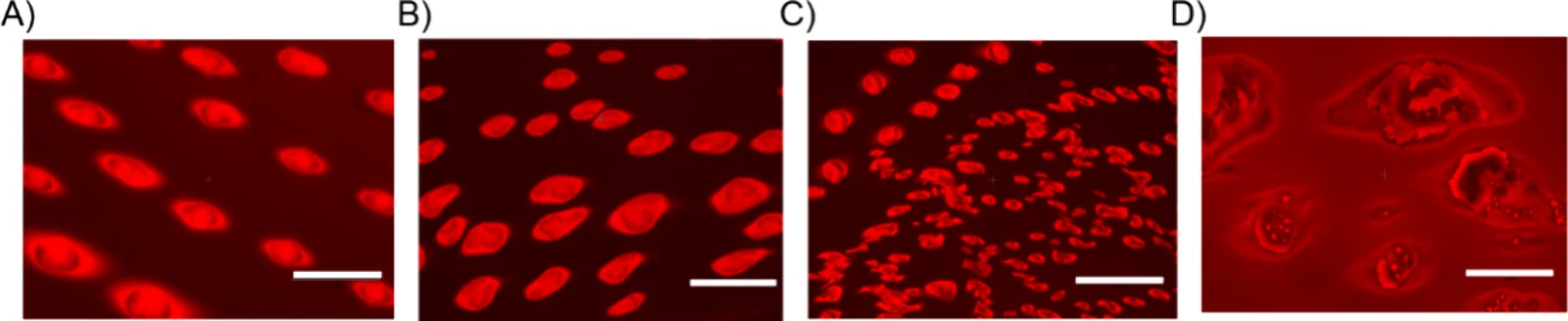
Fluorescence microscopy images of DPTAP and DPTAP: valinomycin monolayers at ∼ 30 mN/m. (A) Pure DPTAP monolayer immediately after solvent evaporation. (B) Pure DPTAP monolayer 30 min after formation. (C) 4:1 DPTAP: valinomycin monolayer prepared by subphase injection, imaged 20 min after injection. (D) 4:1 DPTAP: valinomycin monolayer prepared by cospreading, imaged 20 min after solvent evaporation. Bright regions correspond to liquid expanded phase domains where the fluorescent probe partitions preferentially; dark regions correspond to liquid condensed phase domains. All monolayers contained 0.5 mol % Texas Red DHPE (TR-DHPE) as a fluorescent marker. The scale bar displayed in all panels measures 150 μm.

To determine if the observed differences result from varying amounts of valinomycin at the interface due to different preparation methods, surface pressure vs time measurements were conducted (Figure S1). The surface pressure of a cospread monolayer of DPTAP: valinomycin mixture formed at 30 mN/m remained stable as a function of time; a second monolayer formed by injection of valinomycin into the subphase attained the same surface pressure within 20 min, indicating that the same amount of valinomycin was present at both monolayer interfaces. Despite similar surface pressures, the significant differences in the overall phase behavior of the two DPTAP: valinomycin monolayers in Figures 3C & D indicate that valinomycin must be present at both interfaces but exhibiting different levels of incorporation into the monolayers.

### Molecular-Level Differences in Valinomycin Organization

To identify the molecular details of valinomycin partitioning to the monolayer/water interface in the two different preparation methods, vibrational SFG spectral measurements (Figure 4A) were performed. Figure 4B shows the SFG spectrum of a pure valinomycin monolayer, formed at the air/water interface, in the carbonyl stretching region measured using SSP (S- sum frequency, S- visible, and P-IR) polarization combination. The ester carbonyl modes of pure valinomycin monolayer at 23 mN/m give rise to a broad peak centered around 1760 cm^–1^, consistent with the characteristic C = O stretching mode of the lactone groups.^25,68,69^ In addition, a broad feature centered around 1650 cm^–1^ was observed, arising from the amide I vibrations of the peptide backbone.^68–70^ The same amount of valinomycin required to form a monolayer with a surface pressure of 23 mN/m was then injected into the subphase. The SFG spectrum recorded after an equilibration time of 20 min had almost identical spectral features and amplitude as compared to the drop-cast valinomycin monolayer (Spectral fits in SI section S5). These results indicate that both methods of preparation result in the same monolayer structure of valinomycin at the interface in the absence of a lipid monolayer.

**Figure 4:**
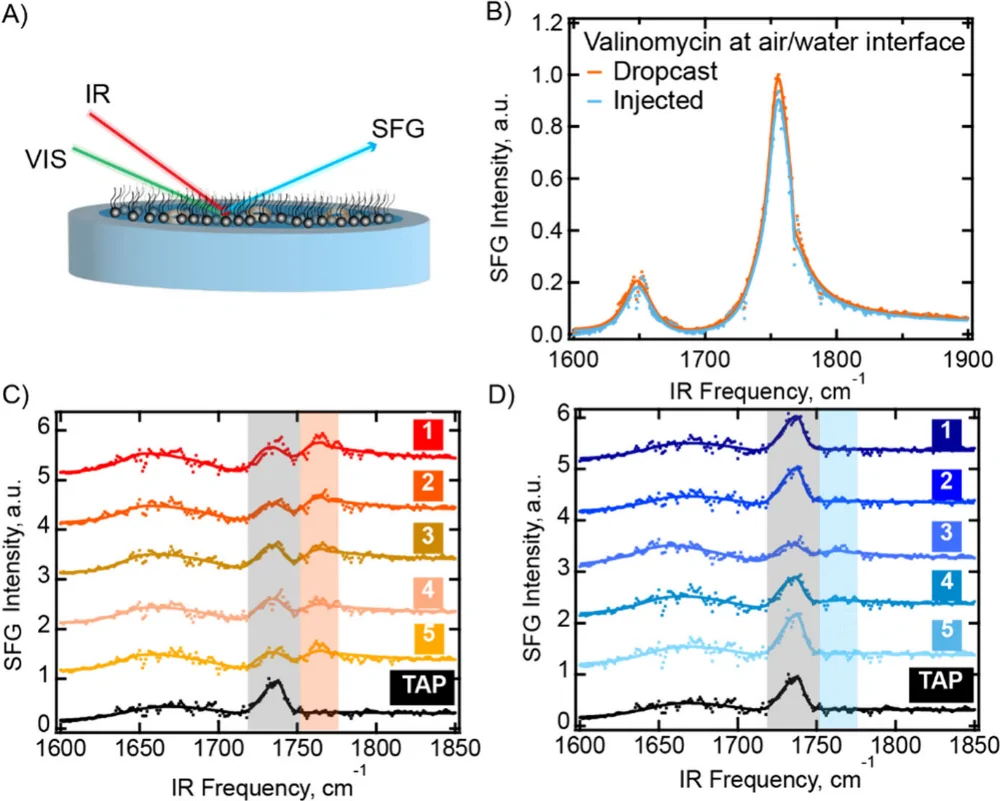
Vibrational sum frequency generation (SFG) spectroscopy of valinomycin and valinomycin-lipid monolayers in the carbonyl stretch region. (A) Schematic of the SFG measurement geometry at the air/water interface. (B) SFG spectra in the carbonyl stretch region of pure valinomycin monolayers formed by spreading from organic solvent (orange) and by injection into the subphase (cyan); surface pressure was 23 mN/m for both samples, chosen as a point that lies sufficiently below collapse as seen from Figure 2A inset. (C, D) SFG spectra of 4:1 DPTAP: valinomycin monolayers prepared by cospreading (C) and injection (D) at a surface pressure of 30 mN/m. Numbered spectra (1–5) were collected from different spatial locations across each monolayer; the bottom spectrum (labeled TAP) shows pure DPTAP for reference. Shaded regions highlight the lipid ester carbonyl and the valinomycin ester carbonyl spectral regions. Spectra in (C) and (D) are vertically offset for clarity. Dots represent data points; solid lines are spectral fits (Spectral fitting details in SI section S5).

The DPTAP lipid monolayers were formed around ∼ 30 mN/m on pure water subphase to characterize the lipid-specific vibrational modes. The black spectrum in Figure 4C represents the carbonyl stretch region of a pure DPTAP monolayer. The peak centered around ∼ 1735 cm^–1^ was assigned to the ester carbonyl of the lipid, and was decomposed into two carbonyl stretch modes at 1723 and 1740 cm^–1^ by fitting.^71–73^ Additionally, the SFG spectrum of pure DPTAP monolayer showed a broad feature between 1600 and 1700 cm^–1^ arising from the interfacial water bending mode.^74–77^ When valinomycin and DPTAP lipids were cospread at the air/water interface, the carbonyl stretch region exhibited two distinct peaks: The 1735 cm^–1^ peak, consisting of two underlying carbonyl populations, originating from the lipid carbonyl modes (Figure 4C, gray shaded region, SI Table S2) and the broad peak around 1760 cm^–1^ peak, consisting of three underlying valinomycin ester carbonyl modes (Figure 4C, orange shaded region, SI Table S2). The appearance of both carbonyl populations indicates that both lipid and valinomycin are present within the monolayer. The amide I band from valinomycin was not visible in this spectrum due to its overlap with the predominant water bending modes in the same vibrational region. SFG spectra were then collected from multiple spots on the monolayer surface to assess the uniformity of valinomycin incorporation (Figure 4C, spectra labeled 1–5). Spectra collected from all spots had a consistent appearance of the valinomycin ester carbonyl modes, indicating that valinomycin is uniformly incorporated throughout the cospread monolayer. In contrast, the injected monolayer (Figure 4D) showed clear spatial heterogeneity. All the probed spots on the mixed monolayer system had the lipid carbonyl peak around 1735 cm^–1^, but the valinomycin ester carbonyl modes were either very weak or absent. Spectral fitting (Table S3) revealed that spectra labeled 1 and 2 in Figure 4D did not require valinomycin carbonyl peaks to fit the spectra, while spectra 3–5 required marginal contributions from valinomycin peaks. The amplitudes of valinomycin peaks in injected monolayer spectra were considerably lower than in the cospread monolayers (Table S4). The lower amplitudes of the valinomycin peaks in conjunction with their nonuniform appearance demonstrated that the injection of valinomycin below a preformed lipid monolayer led to a spatially heterogeneous incorporation.

There are several possible reasons for the low amplitude of valinomycin observed in the injected monolayers. One possibility is that injection might result in valinomycin adsorbing as aggregates below the monolayer surface. If such aggregates form centrosymmetric structures, valinomycin’s dipolar contributions might cancel out. The second possibility is that valinomycin incorporates into the lipid monolayer, but as separate large phase domains. In this scenario, one might observe valinomycin’s SFG response only if the beams probe the specific regions of the monolayer where valinomycin is present.

To gain further insight into these scenarios, SFG spectra were acquired in the C–H and O–H stretch regions (Figure 5). The C–H stretch region reports on specific C–H modes from both lipids and valinomycin, and the O–H stretch region reports on the changes in hydrogen bonding strength and orientational ordering of interfacial water induced by the presence of valinomycin in or near the monolayer. The black spectrum in Figure 5A shows the C–H stretch modes of a pure DPTAP lipid monolayer on water, including contributions from the methylene symmetric stretch (CH_2__-__ss_, 2850 cm^–1^), terminal methyl symmetric stretch (CH_3_-ss, 2874 cm^–1^), and their respective Fermi resonances with the overtone of bending modes (CH_2‑FR_, 2920 cm^–1^; CH_3‑FR_, 2935 cm^–1^)^40,78,79^ Both the terminal CH_3_-as and the headgroup methyl symmetric stretches are expected to contribute around 2960 cm^–1^, but these features and the Fermi resonance contributions interfere with the O–H stretch modes extending into this frequency region, resulting in a prominent dip feature around 2951 cm^–1^.^80,81^ In cospread monolayers of DPTAP: valinomycin, both lipids and valinomycin contribute to the CH_3_-ss and CH_3_-_FR_ peak intensities. However, the increased intensity around 2906 cm^–1^ in mixed monolayers can be uniquely attributed to the valinomycin methine stretch modes (Table S5).^79^ The consistent presence of the valinomycin methine stretch mode across different spots on the cospread monolayer agrees well with the data obtained in the carbonyl region (Figure 4C), indicating that valinomycin is uniformly distributed across the monolayer when cospread.

**Figure 5:**
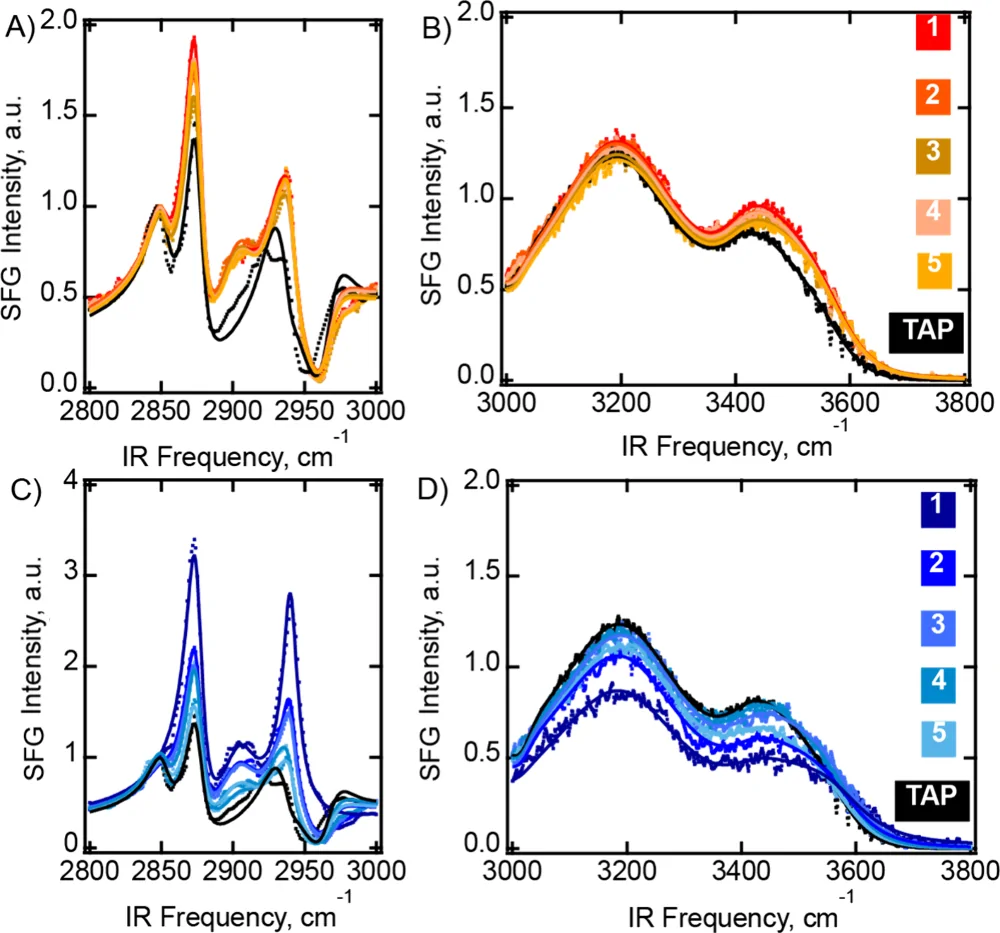
Vibrational sum frequency generation (SFG) spectroscopy of valinomycin-lipid monolayers in the C–H and O–H stretch regions. SFG spectra of 4:1 DPTAP: valinomycin at the air/water interface in the C–H stretch (2800–2950 cm^–1^) and O–H stretch (2950–3800 cm^–1^) regions prepared by cospreading (A,B) and by injection (C,D), at surface pressure of 30 mN/m. Numbered spectra (1–5) were collected from different spatial locations across each monolayer; the bottom spectrum (labeled TAP) shows pure DPTAP for reference. Both C–H and O–H spectra were normalized to the intensity at 2850 cm^–1^. Dots represent data points; solid lines are spectral fits.

We then probed the O–H stretch region to characterize how the surface charge-dependent interfacial water ordering is altered in the presence of valinomycin.

All C–H and O–H spectra were normalized to the lipid CH_2__–ss_ intensity at 2850 cm^–1^ to enable comparison of valinomycin surface concentration and interfacial water ordering relative to a constant lipid reference. The overall intensity in the O–H stretch region (Figure 5B) reports on the extent of anisotropically ordered interfacial water molecules. The pure DPTAP monolayer (Figure 5B, black) exhibited a continuum of O–H stretch modes with two prominent peaks around 3200 cm^–1^ and 3450 cm^–1^. These O–H stretch modes include both water molecules that are directly next to the lipid headgroups as well as water molecules in subsequent layers ordered by the static interfacial electric field.^48,82–85^ Consistent with the behavior in the C–H stretch region, the O–H stretch spectrum of the cospread 4:1 DPTAP: valinomycin monolayers exhibited almost identical spectral shapes and relative intensities across the monolayer (Figure 5B, curves labeled 1–5, Table S5). In contrast, mixed monolayers formed by injection exhibited significant variations across the monolayer. Spot 1 on the injected monolayer (Figure 5D, Table S6) had the maximum amplitude of valinomycin methine peak around 2906 cm^–1^ and the least amplitudes of O–H stretch peak around 3200 cm^–1^ compared to injected monolayer spots 2–5. The amplitudes of both C–H and O–H peaks varied across the injected monolayer (Table S6). The relatively low occurrence of the valinomycin ester carbonyl peak (Figure 4D) agrees with the trends observed in the CH and O–H region of the injected monolayers. Together, they indicate a spatially heterogeneous incorporation of valinomycin in injected monolayers. Further specifics on the peptide incorporation in injected monolayers, and whether aggregates are the reason for the observed heterogeneity, will be investigated in a future study.

## Discussion and Conclusions

The combined isotherm, fluorescence, and SFG data converge on a unified picture: the method used to incorporate valinomycin into a lipid monolayer determines not only the macroscopic phase behavior and lateral homogeneity of the film, but also the molecular location and organizational state of valinomycin within the membrane-mimetic interface. Co-spreading yields a thermodynamically distinct, more homogeneous monolayer in which valinomycin is uniformly distributed and inserted into the acyl chain region of the lipid film. Injection produces a kinetically arrested, phase-separated state in which valinomycin resides heterogeneously at the headgroup-water interface, interacting with but not fully incorporated into the preformed monolayer. In the 28–36 mN/m region probed by the compressional modulus, the magnitude of the preparation-method effect scales with lipid headgroup charge: cationic DPTAP > zwitterionic DPPC > anionic DPPA. We attribute this hierarchy to specific ion-dipole interactions between the positively charged trimethylammonium moieties on DPTAP (and the PC headgroup of DPPC) and the carbonyl-lined cavity of valinomycin, which is the primary cation-binding motif of this ionophore.^86,87^ In DPTAP and DPPC monolayers, these favorable electrostatic interactions drive rapid initial adsorption of valinomycin to the headgroup region upon injection, but the kinetic barrier to subsequent penetration into the already-packed acyl chain region results in a metastable, phase-separated interfacial state. When cospread, the same favorable interactions facilitate intermolecular mixing before the film is assembled, enabling valinomycin to reach a more homogeneous distribution. For anionic DPPA, the absence of complementary headgroup interactions limits valinomycin-lipid mixing regardless of preparation method, rendering the two protocols approximately equivalent.

These observations have significant implications for how mixed amphiphile-lipid monolayer systems should be prepared and interpreted across different research contexts. In studies of antimicrobial peptides and drug-membrane interactions, the injection method is routinely used to mimic the physiological process of amphiphile approaching a preformed biological membrane from the aqueous phase. Based on our results, such experiments will certainly capture the initial adsorption step. However, whether the amphiphile mixes with membrane components is determined by the specific lipid headgroup-amphiphile interactions. Conversely, for applications where the amphiphiles are allowed to preorganize with the membrane components (such as ion selective electrodes and liposome formulations for valinomycin), the cospread Langmuir monolayers will serve as a more accurate model system. The comparisons between studies that employ different preparation methods must therefore be made with caution.

The time scale of our injection experiments allowed a 20 min equilibration time well sufficient for surface pressure saturation. These time scales are relevant for studies involving Langmuir monolayers and other model membrane systems where rapid characterization is desired. However, it is important to note here that very long equilibration times on the scale of several hours might allow more extensive interaction^88^ between injected valinomycin and preformed DPTAP monolayer. Charged lipid monolayers have been shown to undergo a slow relaxation process over extended time scales.^89^ Therefore, it is conceivable that extending the equilibration times would allow valinomycin to gradually insert and undergo rearrangements within the monolayer. We did not systematically investigate this possibility in the present work, and the question of whether the injected state represents a true metastable minimum or simply a long-lived transient remains open.

In conclusion, we have demonstrated that the two different methods of incorporating valinomycin into a Langmuir monolayer of lipids produce fundamentally different molecular arrangements. Co-spreading yields a uniform distribution of valinomycin across the monolayer, whereas injection yields a heterogeneous distribution consistent with kinetically trapped, phase-separated domains. These differences were the most pronounced for cationic lipids and diminished for anionic lipids, suggesting an electrostatic contribution to the incorporation pathway. While different preparation methods are essential in Langmuir monolayers to match their relevant biological or technological context, these findings highlight that the preparation history is a critical parameter that determines the molecular organization within Langmuir monolayers.

## Supplementary Material



## Notes

Experimental section: materials, surface pressure–area measurements, thermodynamic analysis of mixing, SFG measurements, spectral fitting, effect of the organic solvent on the injected monolayer, fluorescence microscopy of the monolayers, Figure S1–S2, Tables S1–S7 (PDF)

## ‡

Department of Chemistry, Birla Institute of Technology and Sciences Pilani Hyderabad Campus, Hyderabad, Telangana, 500078, India

## References

[1] Alberts, B. ; Johnson, A. ; Lewis, J. ; Walter, P. ; Raff, M. ; Roberts, K. Molecular Biology of the Cell 4th ed.: International Student ed.; Routledge, 2002.

[2] Gennis, R. B. Biomembranes: Molecular Structure and Function; Springer, 1989.

[3] Israelachvili, J. N. Intermolecular and Surface Forces; Academic Press, 2011.

[4] Zasloff M. (2002). Antimicrobial
peptides of multicellular organisms. Nature.

[5] Hancock R.
E. W., Sahl H.-G. (2006). Antimicrobial
and host-defense peptides as new anti-infective
therapeutic strategies. Nat. Biotechnol..

[6] Pressman B.
C. (1976). Biological
Applications of Ionophores. Annu. Rev. Biochem..

[7] Matile S., Vargas Jentzsch A., Montenegro J., Fin A. (2011). Recent synthetic transport
systems. Chem. Soc. Rev..

[8] Seddon A. M., Casey D., Law R. V., Gee A., Templer R. H., Ces O. (2009). Drug interactions with lipid membranes. Chem.
Soc. Rev..

[9] Dobson P. D., Kell D. B. (2008). Carrier-mediated
cellular uptake of pharmaceutical
drugs: an exception or the rule?. Nat. Rev.
Drug Discovery.

[10] Langmuir I. (1917). THE CONSTITUTION
AND FUNDAMENTAL PROPERTIES OF SOLIDS AND LIQUIDS. II. LIQUIDS.1. J. Am. Chem. Soc..

[11] Kaganer V. M., Möhwald H., Dutta P. (1999). Structure and phase transitions in
Langmuir monolayers. Rev. Mod. Phys..

[12] Stefaniu C., Brezesinski G., Möhwald H. (2014). Langmuir monolayers as models to
study processes at membrane surfaces. Adv. Colloid
Interface Sci..

[13] Marsh D. (1996). Lateral pressure
in membranes. Biochim. Biophys. Acta.

[14] Brockman H. (1999). Lipid monolayers:
why use half a membrane to characterize protein-membrane interactions?. Curr. Opin. Struct. Biol..

[15] Gálvez-Ruiz M.
J. (2017). Different
approaches to study protein films at air/water interface. Adv. Colloid Interface Sci..

[16] Glomm W. R., Volden S., Halskau Ø., Ese M.-H. G. (2009). Same
System- Different Results: The Importance of Protein-Introduction
Protocols in Langmuir-Monolayer Studies of Lipid-Protein Interactions. Anal. Chem..

[17] Verger R., Pattus F. (1982). Lipid-protein interactions
in monolayers. Chem. Phys. Lipids.

[18] Gaines G. L. (1966). Thermodynamic relationships for mixed insoluble monolayers. J. Colloid Interface Sci..

[19] Dynarowicz-Łątka P., Kita K. (1999). Molecular interaction in mixed monolayers at the air/water interface. Adv. Colloid Interface Sci..

[20] MacRitchie F., Alexander A. E. (1963). Kinetics
of adsorption of proteins at interfaces. Part
I. The role of bulk diffusion in adsorption. J. Colloid Sci..

[21] Graham D. E., Phillips M. C. (1979). Proteins at liquid interfaces: I.
Kinetics of adsorption
and surface denaturation. J. Colloid Interface
Sci..

[22] Bühlmann P., Pretsch E., Bakker E. (1998). Carrier-Based Ion-Selective
Electrodes
and Bulk Optodes. 2. Ionophores for Potentiometric and Optical Sensors. Chem. Rev..

[23] Andreoli T. E., Tieffenberg M., Tosteson D. C. (1967). The Effect of Valinomycin on the
Ionic Permeability of Thin Lipid Membranes. J. Gen. Physiol..

[24] Brockmann H., Schmidt-Kastner G. (1955). Valinomycin
I, XXVII. Mitteil. über Antibiotica
aus Actinomyceten. Chem. Ber..

[25] Shemyakin M. M., Ovchinnikov Y. A., Ivanov V. T., Antonov V. K., Vinogradova E. I., Shkrob A. M., Malenkov G. G., Evstratov A. V., Laine I. A., Melnik E. I., Ryabova I. D. (1969). Cyclodepsipeptides
as chemical tools for studying ionic transport through membranes. J. Membr. Biol..

[26] Tran T. N. T., Qiu S., Chung H.-J. (2018). Potassium
ion selective electrode
using polyaniline and matrix-supported ion-selective PVC membrane. IEEE Sens. J..

[27] Andreoli T. E., Tieffenberg M., Tosteson D. C. (1967). The effect of valinomycin on the
ionic permeability of thin lipid membranes. J. Gen. Physiol..

[28] Stark G., Benz R. (1971). The transport of potassium
through lipid bilayer membranes by the
neutral carriers valinomycin and monactin. J.
Membr. Biol..

[29] Benz R., Stark G., Janko K., Läuger P. (1973). Valinomycin-mediated
ion transport through neutral lipid membranes: Influence of hydrocarbon
chain length and temperature. J. Membr. Biol..

[30] Caspers J., Landuyt-Caufriez M., Deleers M., Ruysschaert J. M. (1979). The effect
of surface charge density on valinomycin-K+ complex formation in model
membranes. Biochim. Biophys. Acta - Biomembr..

[31] Pathirana S., Neely W. C., Myers L. J., Vodyanoy V. (1992). Interaction of valinomycin
and stearic acid in monolayers. Langmuir.

[32] Schifferer F., Cordroch W., Beitinger H., Miibius D., Rahmann H. (1991). Evidence for
the Presence of a Specific Ganglioside GM1/Valinomycin Complex in
Mixed Monolayers. J. Biochem..

[33] Zaitsev S. Y., Zubov V. P., Möbius D. (1995). Mixed monolayers of valinomycin and
dipalmitoylphosphatidic acid. Colloids Surf.
Physicochem. Eng. Aspects.

[34] Shen Y. (1989). Surface properties
probed by second-harmonic and sum-frequency generation. Nature.

[35] Eisenthal K. B. (1996). Liquid
Interfaces Probed by Second-Harmonic and Sum-Frequency Spectroscopy. Chem. Rev..

[36] Richmond G. L. (2002). Molecular
Bonding and Interactions at Aqueous Surfaces as Probed by Vibrational
Sum Frequency Spectroscopy. Chem. Rev..

[37] Chen X., Chen Z. (2006). SFG studies on interactions
between antimicrobial peptides and supported
lipid bilayers. Biochim. Biophys. Acta - Biomembr..

[38] Ishiyama T., Imamura T., Morita A. (2014). Theoretical Studies of Structures
and Vibrational Sum Frequency Generation Spectra at Aqueous Interfaces. Chem. Rev..

[39] Guyot-Sionnest P., Hunt J. H., Shen Y. R. (1987). Sum-frequency
vibrational spectroscopy
of a Langmuir film: Study of molecular orientation of a two-dimensional
system. Phys. Rev. Lett..

[40] Ma G., Allen H. C. (2006). DPPC Langmuir Monolayer
at the Air-Water Interface:
Probing the Tail and Head Groups by Vibrational Sum Frequency Generation
Spectroscopy. Langmuir.

[41] Mashaghi A., Mashaghi S., Reviakine I., Heeren R. M., Sandoghdar V., Bonn M. (2014). Label-free characterization
of biomembranes: from structure to dynamics. Chem. Soc. Rev..

[42] Mondal J. A., Nihonyanagi S., Yamaguchi S., Tahara T. (2010). Structure and Orientation
of Water at Charged Lipid Monolayer/Water Interfaces Probed by Heterodyne-Detected
Vibrational Sum Frequency Generation Spectroscopy. J. Am. Chem. Soc..

[43] Liljeblad J. F. D., Bulone V., Tyrode E. C., Johnson M. C., Rutland M. W. (2012). Molecular
Structure and Stability of Phospholipid Monolayers Probed by Vibrational
Sum Frequency Spectroscopy (VSFS). Biophys.
J..

[44] Ma G., Allen H. C. (2007). Condensing Effect
of Palmitic Acid on DPPC in Mixed
Langmuir Monolayers. Langmuir.

[45] Sovago M., Wurpel G. W. H., Smits M., Müller M., Bonn M. (2007). Calcium-Induced Phospholipid Ordering
Depends on Surface Pressure. J. Am. Chem. Soc..

[46] Inoue K.-i., Takada C., Wang L., Morita A., Ye S. (2020). In Situ Monitoring
of the Unsaturated Phospholipid Monolayer Oxidation in Ambient Air
by HD-SFG Spectroscopy. J. Phys. Chem. B.

[47] Zhang B., Tan J., Li C., Zhang J., Ye S. (2018). Amide I SFG spectral
line width probes the lipid-peptide and peptide-peptide interactions
at cell membrane in situ and in real time. Langmuir.

[48] Pullanchery S., Yang T., Cremer P. S. (2018). Introduction of positive charges
into zwitterionic phospholipid monolayers disrupts water structure
whereas negative charges enhances it. J. Phys.
Chem. B.

[49] Sun S., Sendecki A. M., Pullanchery S., Huang D., Yang T., Cremer P. S. (2018). Multistep interactions between ibuprofen and lipid
membranes. Langmuir.

[50] Ding B., Panahi A., Ho J.-J., Laaser J. E., Brooks C. L., Zanni M. T., Chen Z. (2015). Probing site-specific
structural information of peptides at model membrane interface in
situ. J. Am. Chem. Soc..

[51] Golbek, T. W. ; Franz, J. ; Elliott Fowler, J. ; Schilke, K. F. ; Weidner, T. ; Baio, J. E. Identifying the selectivity of antimicrobial peptides to cell membranes by sum frequency generation spectroscopy. Biointerphases 2017, 12 (2). 10.1116/1.4982710 28476090

[52] Hosseinpour S., Roeters S. J., Bonn M., Peukert W., Woutersen S., Weidner T. (2020). Structure and dynamics
of interfacial peptides and
proteins from vibrational sum-frequency generation spectroscopy. Chem. Rev..

[53] Yan E. C. Y., Fu L., Wang Z., Liu W. (2014). Biological Macromolecules
at Interfaces Probed by Chiral Vibrational Sum Frequency Generation
Spectroscopy. Chem. Rev..

[54] Link K. A., Hsieh C.-Y., Tuladhar A., Chase Z., Wang Z., Wang H., Walker R. A. (2018). Vibrational
studies of saccharide-induced
lipid film reorganization at aqueous/air interfaces. Chem. Phys..

[55] Wang J., Even M. A., Chen X., Schmaier A. H., Waite J. H., Chen Z. (2003). Detection of Amide
I Signals of Interfacial Proteins in Situ Using
SFG. J. Am. Chem. Soc..

[56] Chatterjee S., Maltseva D., Kan Y., Hosseini E., Gonella G., Bonn M., Parekh S. H. (2022). Lipid-driven
condensation and interfacial
ordering of FUS. Sci. Adv..

[57] Biswas B., Roy S., Mondal J. A., Singh P. C. (2020). Interaction of α-Synuclein
with Phospholipids and the Associated Restructuring of Interfacial
Lipid Water: An Interface-Selective Vibrational Spectroscopic Study. Angew. Chem..

[58] Xiao D., Fu L., Liu J., Batista V. S., Yan E. C. Y. (2012). Amphiphilic Adsorption
of Human Islet Amyloid Polypeptide Aggregates to Lipid/Aqueous Interfaces. J. Mol. Biol..

[59] Olenick L. L., Troiano J. M., Vartanian A., Melby E. S., Mensch A. C., Zhang L., Hong J., Mesele O., Qiu T., Bozich J., Lohse S., Zhang X., Kuech T. R., Millevolte A., Gunsolus I., McGeachy A. C., Doğangün M., Li T., Hu D., Walter S. R., Mohaimani A., Schmoldt A., Torelli M. D., Hurley K. R., Dalluge J., Chong G., Feng Z. V., Haynes C. L., Hamers R. J., Pedersen J. A., Cui Q., Hernandez R., Klaper R., Orr G., Murphy C. J., Geiger F. M. (2018). Lipid Corona
Formation from Nanoparticle Interactions with Bilayers. Chem..

[60] Kemp G., Wenner C. E. (1972). Interaction of valinomycin with cations
at the air-water
interface. Biochim. Biophys. Acta - Biomembr..

[61] Zaitsev S. Y., Zubov V. P., Möbius D. (1993). Monolayer
characteristics of valinomycin
in the presence of various salts in aqueous subphase. Biochim. Biophys. Acta - Biomembr..

[62] Marsh, D. Handbook of lipid bilayers; CRC Press, 1990. 10.1201/b11712

[63] Howarth, V. ; Petty, M. ; Lvov, Y. Structure of valinomycin/fatty acid langmuir-blodgett films. In Makromolekulare Chemie. Macromolecular Symposia, 1991; Wiley Online Library: Vol. 51, pp 175–182.

[64] Can S. Z., Chang C. F., Walker R. A. (2008). Spontaneous formation of DPPC monolayers
at aqueous/vapor interfaces and the impact of charged surfactants. Biochim. Biophys. Acta - Biomembr..

[65] Benvegnu D. J., McConnell H. M. (1992). Line tension
between liquid domains in lipid monolayers. J. Phys. Chem..

[66] McConnell H. M. (1991). Structures
and Transitions in Lipid Monolayers at the Air-Water Interface. Annu. Rev. Phys. Chem..

[67] Knobler C. M., Desai R. C. (1992). Phase Transitions
in Monolayers. Annu. Rev. Phys. Chem..

[68] Howarth V., Petty M., Davies G., Yarwood J. (1989). Infrared studies of
valinomycin-containing Langmuir-Blodgett films. Langmuir.

[69] Su Z., Ran X., Leitch J. J., Schwan A. L., Faragher R., Lipkowski J. (2019). How valinomycin
ionophores enter and transport K+ across model lipid bilayer membranes. Langmuir.

[70] Jackson M., Mantsch H. H. (1991). Valinomycin and
its interaction with ions in organic
solvents, detergents, and lipids studied by Fourier transform IR spectroscopy. Biopolymers.

[71] Lewis R. N., McElhaney R. N., Pohle W., Mantsch H. H. (1994). Components of the
carbonyl stretching band in the infrared spectra of hydrated 1,2-diacylglycerolipid
bilayers: a reevaluation. Biophys. J..

[72] Melcrová A., Pokorna S., Pullanchery S., Kohagen M., Jurkiewicz P., Hof M., Jungwirth P., Cremer P. S., Cwiklik L. (2016). The complex nature
of calcium cation interactions with phospholipid bilayers. Sci. Rep..

[73] Dreier L. B., Bonn M., Backus E. H. (2019). Hydration and orientation of carbonyl
groups in oppositely charged lipid monolayers on water. J. Phys. Chem. B.

[74] Seki T., Yu C.-C., Chiang K.-Y., Tan J., Sun S., Ye S., Bonn M., Nagata Y. (2021). Disentangling
sum-frequency generation
spectra of the water bending mode at charged aqueous interfaces. J. Phys. Chem. B.

[75] Vinaykin M., Benderskii A. V. (2012). Vibrational
Sum-Frequency Spectrum of the Water Bend
at the Air/Water Interface. J. Phys. Chem. Lett..

[76] Kundu A., Tanaka S., Ishiyama T., Ahmed M., Inoue K.-i., Nihonyanagi S., Sawai H., Yamaguchi S., Morita A., Tahara T. (2016). Bend Vibration
of Surface Water Investigated
by Heterodyne-Detected Sum Frequency Generation and Theoretical Study:
Dominant Role of Quadrupole. J. Phys. Chem.
Lett..

[77] Sudera P., Cyran J. D., Bonn M., Backus E. H. G. (2021). Interfacial
Vibrational
Spectroscopy of the Water Bending Mode on Ice Ih. J. Phys. Chem. C.

[78] Watry M. R., Tarbuck T. L., Richmond G. L. (2003). Vibrational
Sum-Frequency Studies
of a Series of Phospholipid Monolayers and the Associated Water Structure
at the Vapor/Water Interface. J. Phys. Chem.
B.

[79] Lu R., Gan W., Wu B.-h., Zhang Z., Guo Y., Wang H.-f. (2005). C-H Stretching
Vibrations of Methyl, Methylene and Methine Groups at the Vapor/Alcohol
(n = 1–8) Interfaces. J. Phys. Chem.
B.

[80] Wang J., Buck S. M., Chen Z. (2002). Sum Frequency Generation Vibrational
Spectroscopy Studies on Protein Adsorption. J. Phys. Chem. B.

[81] Sung W., Seok S., Kim D., Tian C. S., Shen Y. R. (2010). Sum-Frequency
Spectroscopic Study of Langmuir Monolayers of Lipids Having Oppositely
Charged Headgroups. Langmuir.

[82] Gonella G., Lütgebaucks C., de Beer A. G. F., Roke S. (2016). Second Harmonic and
Sum-Frequency Generation from Aqueous Interfaces Is Modulated by Interference. J. Phys. Chem. C.

[83] Wen Y.-C., Zha S., Liu X., Yang S., Guo P., Shi G., Fang H., Shen Y. R., Tian C. (2016). Unveiling Microscopic
Structures of Charged Water Interfaces by Surface-Specific Vibrational
Spectroscopy. Phys. Rev. Lett..

[84] Tetteh N., Parshotam S., Gibbs J. M. (2024). Separating Hofmeister Trends in Stern
and Diffuse Layers at a Charged Interface. J.
Phys. Chem. Lett..

[85] Jena K. C., Covert P. A., Hore D. K. (2011). The Effect
of Salt on the Water Structure
at a Charged Solid Surface: Differentiating Second- and Third-order
Nonlinear Contributions. J. Phys. Chem. Lett..

[86] Neupert-Laves K., Dobler M. (1975). The Crystal
Structure of a K+ Complex of Valinomycin. Helv.
Chim. Acta.

[87] Duax W. L., Hauptman H., Weeks C. M., Norton D. A. (1972). Valinomycin
Crystal
Structure Determination by Direct Methods. Science.

[88] Handa T., Nakagaki M. (1979). Miscibility of phospholipids and cholesteryl acetate
in mixed monolayers on aqueous surfaces. Colloid
Polym. Sci..

[89] Viseu M. I., Gonçalves da Silva A. M., Costa S. M. (2001). Reorganization and
desorption of catanionic monolayers. Kinetics of π- t and A-
t relaxation. Langmuir.

