# Supplementary material for: Preparation Method Determines Valinomycin Organization and Phase Behavior in Charged Langmuir Monolayers
Source: J Phys Chem B. 2026 Oct 26;130(44):11809–18. doi: 10.1021/acs.jpcb.6c03977 (PMC13746779; doi:10.1021/acs.jpcb.6c03977)
Supplement: Supplementary file 1 [file jp6c03977_si_001.pdf]

Supporting Information for

Preparation Method Determines Valinomycin Organization and Phase Behavior in Charged Langmuir Monolayers

Amrita Chakraborty[‡1], *Priyojit Das Gupta*[‡,1], *Anabela Meier*[1], *Antara Roy* [1], *Saranya Pullanchery* [1,*]

[1] Department of Chemistry, Texas A&M University, College Station, TX 77843, USA

*Corresponding author: saranya.pullanchery@tamu.edu

S1. Experimental Section: Materials

1,2-dipalmitoyl-sn-glycero-3-phosphocholine (DPPC), 1,2-dipalmitoyl-sn-glycero-3-phosphate (sodium salt) (DPPA), and 1,2-dipalmitoyl-3-trimethylammonium-propane (chloride salt) (DPTAP) were purchased from Avanti Polar Lipids (Alabaster, AL) and were used as received. Texas Red™ 1,2-Dihexadecanoyl-*sn*-Glycero-3-Phosphoethanolamine, Triethylammonium Salt (Texas Red™ DHPE) was purchased from Invitrogen (Carlsbad, CA). Valinomycin was purchased from Sigma Aldrich (St. Louis, MO). Purified water (18.2 MΩ•cm) for all experiments was obtained from a Milli-Q® Ultrapure water purification system.

S2. Surface pressure-area measurements

Surface pressure-mean molecular area isotherms were measured using a KSV NIMA Langmuir trough (Biolin Scientific) with a total surface area of 273 cm^2 and a subphase volume of 176 mL. Before the experiment, the trough and barriers were thoroughly cleaned with Milli-Q water and ethanol. The surface pressure of Langmuir monolayers at the air-water interface was measured by using the Wilhelmy plate method. Pure water was used as a subphase at the experimental temperature 25 ˚C. To make a mixed monolayer, lipid and valinomycin were dissolved in chloroform to make stock solutions having 1 mg/mL concentration. 10-20 μL of the stock solution was spread on the water subphase in a dropwise fashion using a microliter syringe. The chloroform was allowed to evaporate for 20 minutes, and the monolayer was compressed with a barrier speed of 15 mm/minute. To inject valinomycin into the subphase, valinomycin was dissolved in tetrahydrofuran (THF) to make a 1 mg/mL stock solution, and required volume of this solution was injected into the subphase. An additional 20 minutes were allowed for all injected monolayers to equilibrate. For every measurement, the molar ratio of lipid to valinomycin was 4:1. A volume-

matched dosing protocol was used for all three lipid systems and for both preparation methods, so that the two methods deliver identical quantities of both components; the procedure is described in detail in the SFG measurements section below. In brief, the volume of the 4: 1 lipid: valinomycin stock required to reach a target surface pressure was first determined by co-spreading for the corresponding injection experiment. Only the back-calculated lipid portion of the volume was spread, and the complementary valinomycin portion was injected into the subphase. Because the split between the two stock solutions is set by the molar ratio, the individual volumes were calculated separately for each lipid from its molecular weight and that of valinomycin, so a 4:1 molar ratio was delivered in every case.

S3. Thermodynamic analysis of mixing

Deviations from ideal two-dimensional mixing were quantified from the surface pressure-area isotherms[1]. The excess area of mixing at each surface pressure is defined as

$$A_{exc} = A_{12} - (x_1 A_1 + x_2 A_2) \tag{1}$$

and the excess free energy of mixing as

$$\Delta G_{exc} = N_A \int_0^{\pi} [A_{12} - (x_1 A_1 + x_2 A_2)]\, d\pi \tag{2}$$

where A_{12} is the measured mean molecular area of the mixed monolayer, A_1 and A_2 are the mean molecular area of the pure lipid and pure valinomycin monolayers, respectively, at the same surface pressure, $x_1 = 0.8$ and $x_2 = 0.2$ are the mole fractions of lipid and valinomycin, respectively, corresponding to the 4: 1 lipid: valinomycin mixture, and N_A is Avogadro's number.

Negative values of A_{exc} and ΔG_{exc} indicate area condensation relative to ideal mixing due to attractive interactions between the components.

The upper limit of integration was set by the collapse point of the pure valinomycin isotherm (30.7 mN/m, 51.8 Å^2); above this surface pressure, the ideal-mixing curve cannot be constructed. Values of ΔG_{exc} are therefore reported at two upper limits, 25 and 30.7 mN/m (Table S1); both calculations resulted in similar signs and relative magnitudes of ΔG_{exc}.

Table S1. Excess free energy of mixing, ΔG_{exc} (kJ mol^{-1}), for 4:1 lipid: valinomycin. Langmuir monolayers were prepared by co-spreading and by subphase injection, evaluated at two upper limits of integration

Lipid	Preparation	ΔG_{exc} (25 mN m^{-1})	ΔG_{exc} (30.7 mN m^{-1})
DPPA	co-spread	−1.32	−1.60
	injected	+0.78	+0.87
DPPC	co-spread	−2.48	−2.93
	injected	−2.61	−2.89
DPTAP	co-spread	−2.08	−2.21
	injected	−0.19	−0.01

All co-spread monolayers exhibited negative values of ΔG_{exc}, indicating that interactions between lipid and valinomycin are attractive for all three systems when components are pre-mixed. For DPTAP-valinomycin monolayers, which showed maximum difference between injection and co-spreading in the monolayer-bilayer equivalence region, the calculated excess free energy values are close to zero under injection. This implies that injection of valinomycin either leads to ideal mixing or immiscible monolayers with DPTAP. The thermodynamic measurements themselves cannot distinguish between these two scenarios.

We note that ΔG_{exc} is a cumulative parameter, reflecting the behavior over the entire compression below the valinomycin collapse. When comparing the phase behavior of monolayers at 30 mN/m, the compressional modulus provides a better local descriptor to quantify the differences between the two preparation methods. Since these two parameters reflect different pressure regimes and different aspects of mixed films, they need not rank the three lipids in the same order. In the experiments presented here, we performed the spectroscopy and imaging measurements at 30 mN/m, to be at the monolayer-bilayer equivalent surface pressure. The changes in the compressional modulus are better reporters of the differences between the two preparation methods at this surface pressure.

S4. SFG measurements

Vibrational SFG measurements were performed using an SFG spectrometer (SFG092, EKSPLA, Lithuania), consisting of a 1064 nm picosecond Nd: YAG laser system (model PT501, pulse duration 30 ps; 100 Hz repetition rate). The fundamental beam (1064 nm) was split into two, where the majority of the energy was directed towards the generation of mid-IR frequency by difference frequency generation. The remaining 1064 nm fundamental was used to generate the 532 nm visible beam. The fixed frequency visible 532 nm (pulse energy 300 μJ) and tunable IR beams (pulse energy 80 μJ) were overlapped on the sample surface at angles ~60 and ~55° with respect to the surface normal to produce the SFG signal. The incident energies were optimized to prevent laser beam-induced artefacts. The sum frequency signal was collected at an angle of ~59°. The intensity of the sum frequency radiation[2, 3] is proportional to the absolute square of the second-order nonlinear susceptibility, $\chi^{(2)}$:

$$I_{SFG} \propto \left|\chi^{(2)}\right|^2 I_{VIS} I_{IR}$$

where I_{VIS} and I_{IR} are the intensities of the visible and IR beams, respectively. The obtained SFG spectra were normalized with respect to the incident IR and visible intensities. The spectra were collected at 2 cm^{-1} intervals, and each data point was an average of 200 pulses.

SFG measurements were performed using a Teflon petri dish containing a 15 mL aqueous subphase. Monolayers formed in two separate ways: 1) Co-spread monolayers were prepared at the air-water interface by drop casting lipid: valinomycin (4:1) mixtures dissolved in chloroform on the water subphase to achieve a surface pressure of ~30 mN/m. A total of 5 μL of 1 mg/ml stock solutions was required to reach this surface pressure. 2) A pure lipid monolayer was first drop-cast from a chloroform solution and allowed to equilibrate on the subphase. Subsequently, valinomycin solution in tetrahydrofuran (THF) was injected into the subphase. The amount of valinomycin to be injected was calibrated to achieve the same 4:1 (lipid: valinomycin) ratio in both experiments using a volume-matched dosing protocol. From the 4: 1 molar ratio and the molecular weights of DPTAP and valinomycin, the 5 μL total corresponds to 3.5 μL of the lipid stock and 1.5 μL of valinomycin stock. For the injection experiments, only the 3.5 μL lipid portion was drop-cast, giving rise to a pre-injection surface pressure of 0.63 mN/m (Figure S1). 1.5 μL of 1 mg/ml valinomycin in THF was then injected beneath this preformed film, yielding a nominal subphase concentration of 0.1 mg/L (90 nM) and a THF carrier concentration of 0.010% v/v in the 15 mL subphase. The surface pressure subsequently rose to ~ 30 mN/m (Figure S1). Both preparations therefore contain identical quantities of lipid and valinomycin, at molar ratio of 4: 1, and reach the same final surface pressure; they differ only in preparation pathway. To assess the lateral uniformity of the monolayers, spectra were recorded at five spatially distinct locations across each film by translating the sample stage between acquisitions, with the beam overlap re-optimized at

each position. Spectra of a pure lipid monolayer prepared under identical conditions (~ 30 mN/m) were recorded for reference.

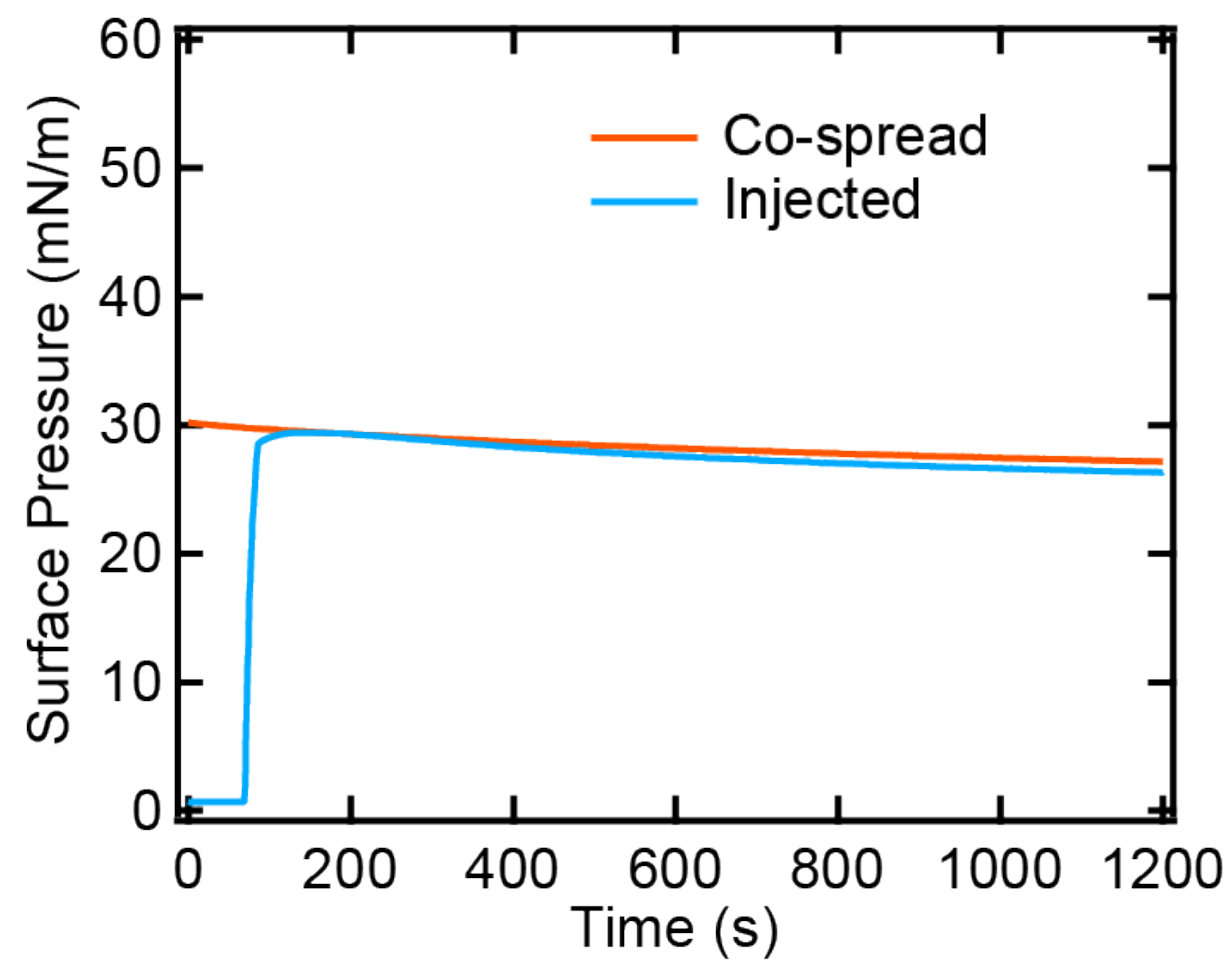

Figure S1. Surface pressure–time profiles of 4:1 DPTAP: valinomycin monolayers prepared by co-spreading and by subphase injection. For the injected film (blue), the back-calculated lipid fraction was first spread alone, giving a pre-injection surface pressure of 0.63 mN/m. The co-spread film (orange) was formed by spreading DPTAP and valinomycin mixture together. Because the two preparations contain identical dosed quantities of both components, their convergence to the same surface pressure indicates equivalent interfacial composition. Both films subsequently show same slow relaxation over time, consistent with the quasi-equilibrium character of these monolayers.

S5. Spectral fitting

The intensity of sum frequency signal follows equation 3:

$$I_{SFG} \propto | \chi_{eff}^{(2)} | \times I_{Vis} \times I_{IR} \tag{3}$$

where I_{Vis} and I_{IR} are the intensities of the incoming visible and infrared laser beams, respectively. $| \chi_{eff}^{(2)} |$ represents the second order nonlinear susceptibility, which can be further expressed as:

$$\chi_{eff}^{(2)} = \chi_{NR}^{(2)} + \chi_{R}^{(2)} = \chi_{NR}^{(2)} + \sum_q \frac{A_q}{\omega_{IR} - \omega_q + i\Gamma_q} \tag{4}$$

where $\chi_{NR}^{(2)}$ and $\chi_{R}^{(2)}$ are the frequency-independent non-resonant susceptibility term and the frequency-dependent resonant susceptibility term, respectively. $\chi_{R}^{(2)}$ of the qth resonant mode is a function of A_q(amplitude), ω_q (resonance frequency), Γ_q (linewidth), and ω_{IR} (frequency of the input infrared laser beam). Spectra were normalized to the intensities of the incoming visible and IR beams. The normalized spectra were then fit to equation (4). The normalized intensity measured in our experiments is proportional to the absolute square of $\chi_{eff}^{(2)}$.

Table S2. Fit parameters for the carbonyl/amide region of the co-spread DPTAP-valinomycin monolayer. over the five spots of the film.

Peak	ω_q(cm^{-1})	Γ_q (cm^{-1})	Co-spread 1	Co-spread 2	Co-spread 3	Co-spread 4	Co-spread 5
H–O–H bend	1650	39.56	42.25	41.47	41.40	39.67	42.69
DPTAP C=O	1723.9	12.62	6.14	4.55	4.87	4.91	4.19
DPTAP C=O	1740.3	6.57	-0.45	-0.56	-1.25	-0.92	-0.46
Valinomycin amide I	1647.4	13.5	1.68	1.62	1.37	0.94	1.14
Valinomycin amide I	1666.0	28.0	-0.93	-0.90	-0.76	-0.52	-0.63
valinomycin ester C=O	1740.7	16.2	1.11	1.07	0.90	0.62	0.75
valinomycin ester C=O	1754.3	9.8	2.28	2.20	1.86	1.27	1.55
valinomycin ester C=O	1767.0	2.0	-0.06	-0.06	-0.05	-0.03	-0.04

Table S3. Fit parameters for the carbonyl/amide region of the injected DPTAP-valinomycin monolayer. over the five spots of the film.

Peak	ω_q(cm^{-1})	Γ_q (cm^{-1})	Injected 1	Injected 2	Injected 3	Injected 4	Injected 5
H–O–H bend	1650	39.56	46.30	44.80	40.97	45.68	47.55
DPTAP C=O	1723.9	12.62	3.71	4.15	4.27	4.55	4.18
DPTAP C=O	1740.3	6.57	-2.64	-2.66	-0.61	-1.87	-2.97
Valinomycin amide I	1647.4	13.5	0	0	0.55	0.27	0.003

Valinomycin amide I	1666.0	28.0	0	0	-0.30	-0.15	-0.001
valinomycin ester C=O	1740.7	16.2	0	0	0.36	0.17	0.002
valinomycin ester C=O	1754.3	9.8	0	0	0.74	0.36	0.004
valinomycin ester C=O	1767.0	2.0	0	0	-0.02	-0.01	-0.0001

Table S4. Fit parameters for the carbonyl/amide region of the co-spread and injected DPTAP-valinomycin monolayer ($A_{NR} = 0.512 \pm 0.04$, $\varphi_{NR} = 1.22 \pm 0.08$). Mean ± Standard deviation (SD) over the five spots of each film.

**Peak**	**ω_q(cm^{-1})**	**Γ_q (cm^{-1})**	**Co-spread (n=5)**	**Injected (n =5)**	**Pure DPTAP (for reference)**	**Pure Valinomycin (for reference)**
H–O–H bend	1650	39.56	41.50 ± 1.15	45.1 ± 2.50	42.9372	-
DPTAP C=O	1723.9	12.62	4.93 ± 0.73	4.17 ± 0.30	3.6961	-
DPTAP C=O	1740.3	6.57	-0.73 ± 0.34	-2.14 ± 0.95	-2.7005	-
Valinomycin amide I	1647.4	13.5	1.35 ± 0.32	0.16 ± 0.24	-	5.12(Dropcast) 5.15(Injected)
Valinomycin amide I	1666.0	28.0	-0.75 ± 0.18	-0.09 ± 0.14	-	-2.84(Dropcast) -3.24(Injected)
valinomycin ester C=O	1740.7	16.2	0.89 ± 0.21	0.11 ± 0.16	-	3.37(Dropcast) 3.51(Injected)
valinomycin ester C=O	1754.3	9.8	1.83 ± 0.43	0.22 ± 0.33	-	6.95(Dropcast) 6.70(Injected)
valinomycin ester C=O	1767.0	2.0	-0.05 ± 0.01	-0.01 ± 0.01	-	-0.18(Dropcast) -0.21(Injected)

Table S5. Fit parameters for the C-H/O-H region of the co-spread DPTAP-valinomycin monolayer over the five spots of each film.

**Peak**	**ω_q(cm^{-1})**	**Γ_q (cm^{-1})**	**Co-spread 1**	**Co-spread 2**	**Co-spread 3**	**Co-spread 4**	**Co-spread 5**
CH_2-ss	2852	8.62	-1.73	-1.97	-1.97	-1.99	-1.96
CH_3-ss	2875	7.01	-5.97	-5.42	-5.12	-5.73	-5.74
Methine C-H	2906.39	17.18	-6.47	-6.85	-6.95	-6.61	-6.85
CH_2- FR	2934	14.94	-18.37	-17.60	-17.26	-17.37	-17.22
CH_3- FR	2940.0	6	13.61	13.41	13.00	13.23	13.25
CH_3-as	2966	12	6.47	6.30	6.10	6.41	6.10
Choline CH_3- ss	2976	16.02	0	0	0	0	0
Choline CH_3- as	3022	28	1.47	1.54	1.49	1.31	1.50
O-H 1	3196.29	158.55	-193.01	-189.04	-188.8	-187.71	-185.28

O-H 2	3412.15	86.07	-34.59	-33.27	-31.56	-32.79	-30.52
O-H 3	3571.84	97.59	58.05	60.40	57.22	59.99	60.5

Table S6. Fit parameters for the C-H/O-H region of the injected DPTAP-valinomycin monolayer over the five spots of each film.

**Peak**	**ω_q(cm^{-1})**	**Γ_q (cm^{-1})**	**Injected 1**	**Injected 2**	**Injected 3**	**Injected 4**	**Injected 5**
CH_2-ss	2852	8.62	0	-0.66	-1.09	-2.26	-2.30
CH_3-ss	2875	7.01	-8.33	-6.24	-5.85	-6.42	-5.09
Methine C-H	2906.39	17.18	-10.66	-8.64	-8.37	-5.07	-6.79
CH_2- FR	2934	14.94	-16.59	-17.73	-17.56	-17.88	-15.12
CH_3- FR	2940.0	6	18.06	15.22	14.75	12.87	12.16
CH_3-as	2966	12	0	4.53	4.87	7.38	5.97
Choline CH_3- ss	2976	16.02	0	0	0	0	0
Choline CH_3- as	3022	28	0.59	2.10	2.09	2.50	2.34
O-H 1	3196.29	158.55	-148.22	-171.03	-181.57	-177.63	-171.46
O-H 2	3412.15	86.07	-21.16	-23.95	-26.12	-30.88	-24.87
O-H 3	3571.84	97.59	70.27	61.99	64.77	60.42	56.23

Note: The injected 1 position differs from the other four positions on the injection film, and the difference is present in the raw spectra as well as the fit results: the amplitudes of C-H and O-H stretch peaks fall well outside the range spanned by injected 2-5. Fitting from several hundred randomized starting points returned the same minimum in every case. Injected 1 thus has the highest amplitude for CH_3-ss, Methine C-H, and a distinct interfacial water signal compared to the other positions, consistent with a locally valinomycin-rich region.

Table S7. Fit parameters for the C-H/O-H region of the co-spread and injected DPTAP-valinomycin monolayer (A_{NR} = 0.15 ± 0.03, φ_{NR} = -0.48 ± 0.25). Mean ± Standard deviation (SD) over the five spots of each film.

Peak	ω_q(cm^{-1})	Γ_q (cm^{-1})	Co-spread (n=5)	Injected (n =5)	Pure DPTAP (for reference)
CH_2-ss	2852	8.62	-1.92 ± 0.11	-1.26 ± 1.01	-2.92
CH_3-ss	2875	7.01	-5.6 ± 0.33	-6.38 ± 1.20	-5.13
Methine C-H	2906.39	17.18	-6.74 ± 0.20	-7.90 ± 2.09	-
CH_2- FR	2934	14.94	-17.56 ± 0.47	-16.98 ± 1.15	-11.74
CH_3- FR	2940.0	6	13.30 ± 0.22	14.61 ± 2.30	1.22
CH_3-as	2966	12	6.27 ± 0.17	4.55 ± 2.77	12.39
Choline CH_3- ss	2976	16.02	0	0	-2.15
Choline CH_3- as	3022	28	1.46 ± 0.09	1.92 ± 0.77	2.96
O-H 1	3196.29	158.55	-188.77 ± 2.80	-169.98 ± 12.94	-168.89
O-H 2	3412.15	86.07	-32.54 ± 1.57	-25.39 ± 3.57	-32.45
O-H 3	3571.84	97.59	59.23 ± 1.50	62.54 ± 5.22	61.33

S6. Effect of organic solvent on the injected monolayer

To form the injected monolayer, valinomycin was introduced as a THF solution beneath the pre-formed lipid monolayer. The volume of injected THF was 1.5 μL in a total subphase volume of 15 mL, corresponding to 0.01% v/v or 1.2 mM THF in the subphase. To investigate the effect of THF alone on the lipid monolayer, the surface pressure of a DPTAP monolayer was monitored after injection of THF into the subphase (Figure S2A).

The surface pressure of a DPTAP monolayer at 30 mN/m was monitored as a function of time. At time, t = 100 s, THF was injected into the subphase (marked with an arrow on Figure S2A). The

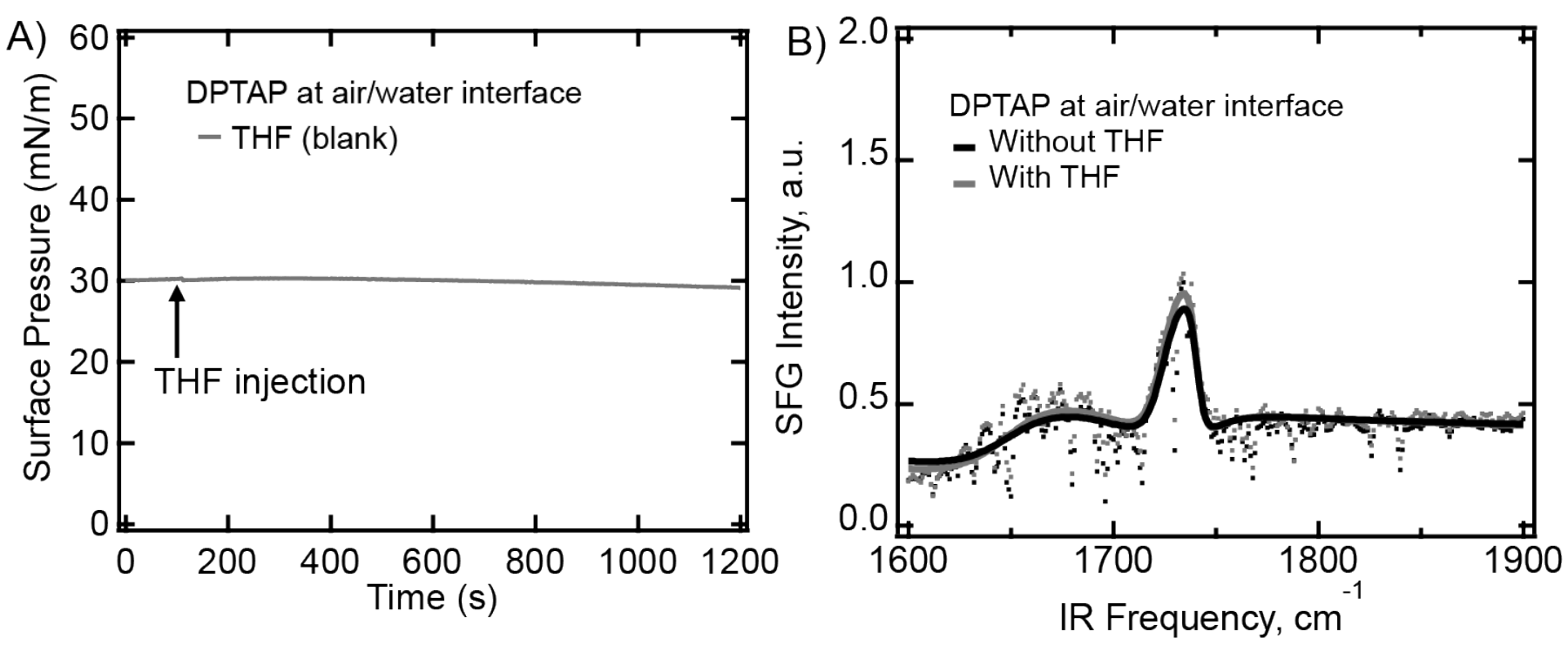

Figure S2. Control experiments to investigate the effect of injected organic solvent on DPTAP monolayers. A) Surface pressure before and after injection of 1.5 μL of neat THF into the subphase beneath the monolayer. THF was injected at t = 100 s. B) Vibrational SFG spectra in the carbonyl stretch region before (black) and after (grey) THF injection, dots are measured intensities and solid lines are fits.

surface pressure remained the same throughout the 20 minutes of measurement, indicating no loss or significant reorganization of molecules at the interface. Vibrational SFG spectra recorded before and after injection of THF are shown in Figure S2B. The spectral shape and intensity remained the same upon injection of THF alone, indicating that THF does not perturb the DPTAP monolayer at this concentration, and the spectral differences reported for the injected DPTAP: valinomycin monolayer arise from valinomycin rather than from the solvent.

S7. Fluorescence microscopy of the monolayers

Fluorescence microscopy experiments were performed at room temperature using an Invitrogen™ EVOS™ M5000 Imaging system (Thermo Fisher Scientific) equipped with a CMOS camera (2048 × 1536-pixel resolution, 3.2 Megapixels). Monolayers were prepared on a transparent petri dish with a subphase volume of 5 mL. Both mixed and injected lipid monolayers were labelled with 0.5 mol% Texas Red DHPE, and the total amount of lipid-valinomycin mixtures was calibrated to maintain identical surface pressures as in the SFG experiments.

References

(1) Jurak, M.; Szafran, K.; Cea, P.; Martín, S. Analysis of molecular interactions between components in phospholipid-immunosuppressant-antioxidant mixed Langmuir films. *Langmuir* **2021**, *37* (18), 5601.
(2) Guyot-Sionnest, P.; Hunt, J. H.; Shen, Y. R. Sum-frequency vibrational spectroscopy of a Langmuir film: Study of molecular orientation of a two-dimensional system. *Physical Review Letters* **1987**, *59* (14), 1597–1600. DOI: 10.1103/PhysRevLett.59.1597.
(3) Shen, Y. Surface properties probed by second-harmonic and sum-frequency generation. *Nature* **1989**, *337* (6207), 519–525.
(4) Backus, E. H.; Bonn, D.; Cantin, S.; Roke, S.; Bonn, M. Laser-heating-induced displacement of surfactants on the water surface. *The Journal of Physical Chemistry B* **2012**, *116* (9), 2703–2712.
